# Bead beating demonstrates enhanced bacterial detection in kidney stone culture compared to traditional mortar-pestle processing

**DOI:** 10.1128/spectrum.00889-25

**Published:** 2025-07-07

**Authors:** Tyler A. On, Farhan Anwar, Austin L. Chien, Chiu-Hsieh Hsu, Gayatri Vedantam, William D. Lainhart, David T. Tzou

**Affiliations:** 1 University of Arizona College of Medicine 12216 , Tucson, Arizona, USA; 2 Animal and Comparative Biomedical Sciences, University of Arizona 8041, Tucson, Arizona, USA; 3 Department of Urology, University of Arizona College of Medicine 12216 , Tucson, Arizona, USA; 4 College of Public Health, University of Arizona 8041, Tucson, Arizona, USA; 5 Department of Pathology and Laboratory Medicine, University of Arizona 8041, Tucson, Arizona, USA; University of Maryland School of Medicine, Baltimore, Maryland, USA

**Keywords:** kidney stone culture, bead beating, mortar-pestle, percutaneous nephrolithotomy (PCNL)

## Abstract

Percutaneous nephrolithotomy (PCNL) is a standard operation for treating large kidney stones but carries a risk of postoperative infection. Recent guidelines recommend obtaining a stone culture or kidney stone culture, yet no standardized protocol exists for processing kidney stones. Bead beating (BB) is a standardizable method of specimen preparation technique, which has been utilized for bacterial culture in other fields, but its application to kidney stone cultures is novel. Sixty kidney stone specimens were collected prospectively from patients undergoing PCNL, and each specimen was processed by either BB or mortar and pestle (MP). Following homogenization, samples were cultured with the standard procedure for a high-quality urine culture. Microorganisms were identified using matrix-assisted laser desorption/ionization time-of-flight mass spectrometry and quantified following standard laboratory procedures. Statistical comparisons were performed using McNemar’s test and paired *t*-tests, as appropriate. Of the 60 kidney stones, 25 (41.7%) yielded microbial growth, with BB detecting growth in all 25 stones compared to MP detecting growth in 18 (*P* = 0.02). Both methods identified the same microorganisms in 17 stones, while BB uniquely detected organisms in seven stones, and MP uniquely detected *Proteus mirabilis* in one stone. BB demonstrated higher sensitivity for detecting microbial growth compared to MP, suggesting that adopting a standardized protocol incorporating BB could improve diagnostic accuracy and enhance our understanding of infection-related stone disease. Further studies should evaluate the clinical implications of these findings and determine whether BB-based stone culture processing and subsequent culture results correlate with postoperative infection outcomes.

**IMPORTANCE:**

This study provides a rapid and standardized method for kidney stone culture using bead beating, which significantly improves microbial detection compared to traditional mortar-pestle techniques. This method accelerates processing and enhances sensitivity for identifying microbes present in kidney stones, offering the potential for more accurate diagnoses. By providing a robust and efficient protocol, it paves the way for standardized practices across laboratories and clinical settings.

## INTRODUCTION

Percutaneous nephrolithotomy (PCNL) is a standard surgical treatment for the removal of large (>2 cm) kidney stones (1), yet it remains associated with a risk for postoperative sepsis (2, 3). Renal calculi can harbor infectious organisms, which can lead to the development of infectious complications despite negative preoperative urine cultures (4, 5). Increasing evidence suggests that intra-operative kidney urine or stone cultures more accurately identify the causative organisms of postoperative infections following PCNL (4, 6). This is reflected in the recent 2023 update to the European Association of Urology (EAU) Urolithiasis Guidelines, which recommend that “urine taken directly from the renal pelvis, or a stone culture be performed at the time of PCNL as they are more predictive of post-PCNL sepsis than preoperative midstream urine culture” (7).

Despite this guideline recommendation, there remains a lack of a standardized microbiology protocol or standard policy recommendation for how to best perform clinical stone cultures. Our group previously demonstrated that among fellowship-trained endourologists, there is a large variability not only in practice patterns, but also in how stone cultures are performed by microbiology labs at multiple institutions (8). The American Society for Microbiology provided a preliminary protocol in the *Clinical Microbiology Procedures Handbook* (5th edition), stating to “grind the stone to a homogeneous consistency using a sterile mortar and pestle (MP)” (9). Grinding for how long and to what extent to produce a “homogeneous consistency” remains unclear.

Bead beating (BB), a method for tissue/specimen homogenization involving rapid shaking of glass or metal beads in a reinforced vial, is generally more standardized and unbiased than other mechanical grinding methods (10). While BB has been used in microbiological preparation in other fields of medicine (11–15), its application to stone cultures is relatively novel. This study aims to determine whether kidney stone fragmentation using BB versus traditional mortar and pestle (MP) results in differences in microbial identification and quantification from stone cultures.

## MATERIALS AND METHODS

### Patient and sample collection

The University of Arizona is a site for Registry for Stones of the Kidney and Ureter (ReSKU), which is an institutional review board-approved prospectively collected database (#1910099958). Patients are enrolled during clinic and inpatient visits by the site principal investigator (DTT). ReSKU then allows for clinical data to be tracked and combined with bio-banked stone and urine samples (14).

At our institution, a preoperative urine culture is obtained from all patients, usually 10–14 days prior to undergoing PCNL. Patients with a positive urine culture are treated with an appropriate preoperative multi-day course of culture-specific antibiotics. This is separate from immediately prior to the start of a PCNL, when patients always receive a dose of i.v. antibiotics as recommended by Guidelines (1). Preoperative cultures direct what i.v. antibiotic the patient receives, and if this culture shows no growth, patients generally receive a second-generation i.v. cephalosporin (e.g., Cefoxitin) immediately prior to the start of their PCNL.

Biospecimens are taken from every PCNL performed by a single surgeon (DTT). With respect to the PCNL technique, a 24-French balloon dilator is used, and every effort is made to fragment stones with a lithotrite (Swiss Lithoclast Trilogy, Boston Scientific) such that the largest fragments can be removed through a 26-French renal sheath. Both intact stones and fragments removed at the time of surgery are placed directly into individual vials containing 1 mL of 30% glycerol/PBS solution, which are then stored at −80°C. For this study, kidney stone fragments were collected from 60 unique PCNL patients taken from PCNL operations that occurred between August 2023 and August 2024. All stone analyses were performed at Quest Diagnostics Nichols Institute in San Juan Capistrano, California.

### Sample preparation

**Fig 1:**
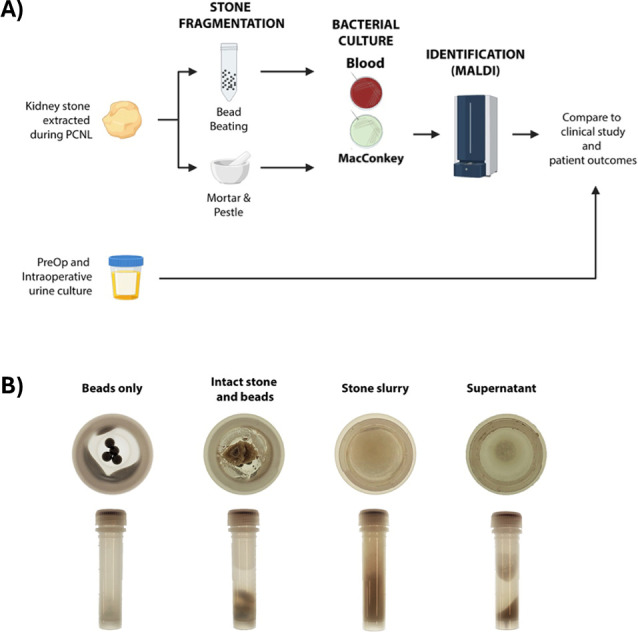
Study design. The study design is outlined in (**A**). Specimens are processed by BB or MP and then plated. Isolated colonies are identified by MALDI-TOF MS and compared to preoperative urine cultures. Examples of bead tubes pre-processing and post-processing are shown in (**B**).

Figure 1 provides a schematic of the overall study design and workflow, as described below.

#### Bead Beating

Fragments from each patient were thawed, weighed, and prepared for bacterial culture. Kidney stone fragments were placed in prefilled metal bead tubes (Omni International) with 1 mL of 30% glycerol/PBS solution. The samples were homogenized using a Bead Ruptor 24 (Omni International, SKU:19-070) for one cycle at 8,000 rpm for 15 seconds. After the slurry was created, the entire tube was then transferred and chilled on ice for 10 minutes to abate the heat generated during the BB step, followed by pelleting with a pulse spin centrifuge. NOTE: This cool-down and pelleting step occurred due to downstream experimentation performed on these specimens for other studies. Though these steps are not needed for microbiology culture, the steps were included here for completeness.

#### Mortar and pestle

For MP processing, samples were thawed, weighed, and ground for 15 seconds using a tissue grinder (Fisher Scientific; SKU:129471). The ground specimen was then pelleted with a pulse spin centrifuge. For both BB and MP, 5% blood agar and McConkey agar plates were inoculated with 10 µL of the supernatant. The plates were streaked for quantification and incubated in 5% CO_2_ at 37°C for up to 72 hours. Individual colonies were identified with matrix-assisted laser desorption/ionization time-of-flight mass spectrometry (MALDI-TOF MS) using the Bruker Biotyper Sirius MALDI-TOF MS (database revision J; Bruker Daltonik GmbH, Billerica, MA).

### Statistical analysis

For comparing BB versus MP results, statistical analysis was done using R version 4.1.0 (R Core Team, Vienna, Austria). To compare stone mass between BB and MP, a paired *t*-test was performed. Discrepancies in organism growth between BB and MP were reported using McNemar’s test to determine statistical significance.

## RESULTS

The demographic features and stone characteristics of the 60 patients are summarized in Table 1. Of the 60 kidney stones cultured, 25 (41.7%) harbored microbes (gram-positive bacteria, gram-negative bacteria, and yeast) with CFU counts ranging from 100 to >100,000 CFU/mL after stone homogenization. No additional organism growth was seen after 48 hours. Among these 60 patients, 40/60 (66.7%) had a preoperative bladder urine culture showing bacteria, with 27/60 (45%) having either a ureteral stent or nephrostomy tube placed prior to their PCNL.

**TABLE 1:** Patient summary and stone characteristics

Parameter	Total	Positive stone culture
Age		
Mean	59.3	58.6
Median	63	64
Range	18–83	18–83
Sex		
Male	29 (48.3%)	14 (56%)
Female	31 (51.7%)	11 (44%)
Recent ureteral stent or nephrostomy tube		
Stent	17/60 (28.3%)	9/25 (36%)
Nephrostomy tube	8/60 (13.3%)	6/25 (24%)
Both	2/60 (3.3%)	1/25 (4%)
Predominant composition >50%		
Calcium oxalate	29/60 (48.3%)	6/25 (24%)
Carbonate apatite	17/60 (28.3%)	11/25 (44%)
Struvite	8/60 (13.3%)	5/25 (20%)
Uric acid	3/60 (5.0%)	0/25 (0%)
Unknown^*a*^	2/60 (3.3%)	2/25 (8%)
Cystine	1/60 (1.7%)	1/25 (4%)
Staghorn calculi		
Staghorn	8/60 (13.3%)	3/25 (12%)
Partial staghorn	16/60 (26.7%)	5/25 (20%)
Stone burden (mm)		
Mean	53.3	49.5
Median	44.5	41
Range	12–195	12–125

^*a*^ Stone analysis report: “After repeated analysis, we were unable to identify the constituents of stone”.

**Fig 2:**
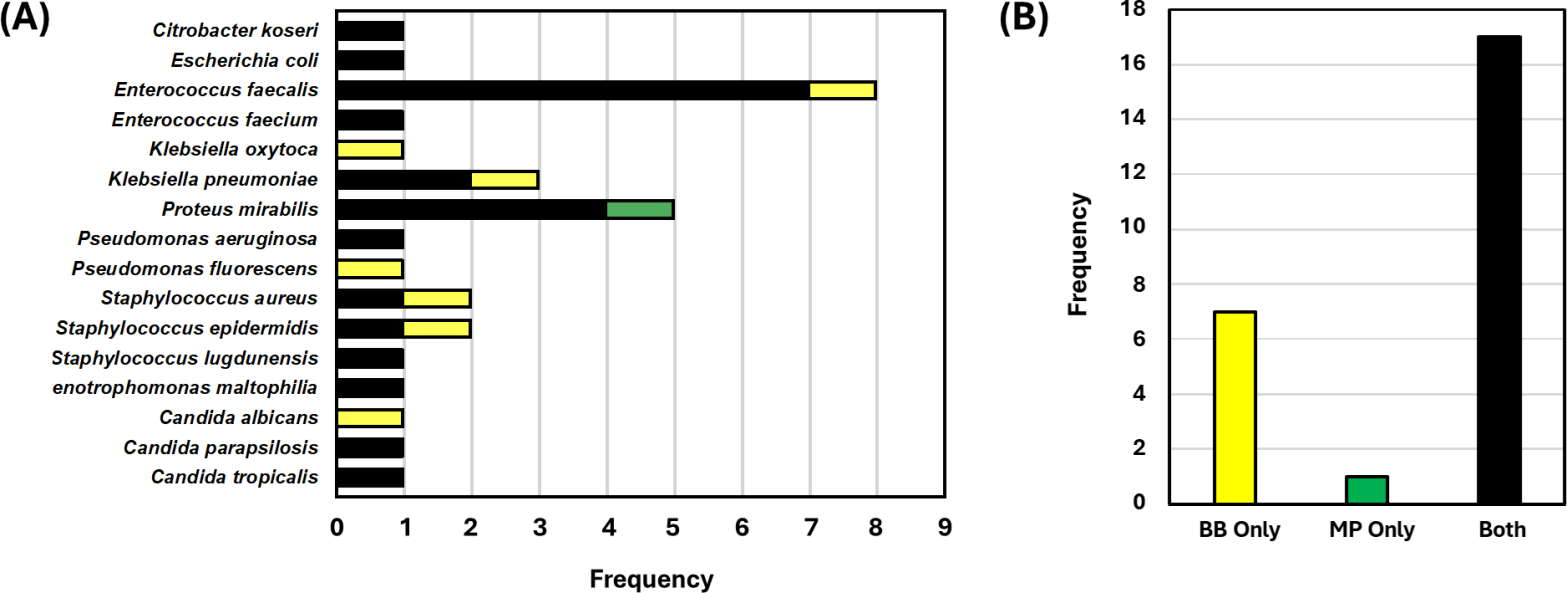
Microorganisms identified using both bead beating and mortar-pestle. (**A**) Stone cultures on kidney stone specimens resulted in both gram-positive and gram-negative bacteria as well as *Candida* sp. Black bars indicate species identified in both BB and MP. Green indicates species isolated from MP only. Yellow indicates species isolated from BB only. (**B**) While both BB and MP identified the same microorganism in 17 stones, BB demonstrated exclusive growth in seven stones.

All patients received immediate preoperative antibiotics as recommended by AUA Guidelines (1). Among the 25 patients with stones that harbored microbes on stone culture, 19 (76%) had received a preoperative course of antibiotics based on the bacteria and sensitivities found on a preoperative urine culture. In 12 (48%) of these 25 stones, the same microbe was identified on stone culture as that found on preoperative bladder urine culture. Meanwhile, 13 (52%) had the same microbe found on stone culture as the intra-operative renal pelvis urine culture. With respect to stone composition, 16 (64.0%) of the 25 stones harboring microbes had compositions traditionally considered infectious (primarily Struvite and Carbonate apatite), while 6 (24.0%) were calcium oxalate stones, and 1 (4.0%) was cystine. Moreover, 8 of the 25 (32%) stones with growth were either staghorn or partial staghorn calculi. A comparison of the organisms recovered from BB versus MP stone culture is depicted in Fig. 2.

Table 2 shows patient culture data comparing organisms recovered from preoperative urine cultures to those from intra-operative kidney urine, intra-operative bladder urine, and kidney stone cultures (note—preoperative and intra-operative urine cultures were processed by our clinical laboratory and reported according to the laboratory’s standard operating procedures). BB successfully recovered microbial growth from 25 stones, compared to 18 stones with MP (Table 2, *P* = 0.02). Both methods identified the same microorganisms in 17 stones. In seven stones, organism growth was detected exclusively in BB-processed specimens. There were five stones for which both BB and MP identified two microbes. In addition, there was a single stone in which both BB and MP identified yeast, but MP also found *Proteus mirabilis*. Table 3 shows the mass of the stones and the organisms detected among those where we observed discordant results between BB- and MP-processed stone specimens. Despite there being a few notable differences in the stone mass of these BB- and MP-processed specimens, this difference was not significant (*P* = 0.26).

**TABLE 2:** Organisms recovered from preoperative and intra-operative urine cultures in patients with positive kidney stone culture

Sample	Preoperative urine culture	Intra-operative kidney urine culture	Intra-operative bladder urine culture	Kidney stone Bead beating	Kidney stone Mortar and pestle
2	*Klebsiella pneumoniae* *Providencia rettgeri*	*Enterococcus faecalis*	No culture performed	*Klebsiella pneumoniae* *Enterococcus faecalis*	*Klebsiella pneumoniae* *Enterococcus faecalis*
5	*Proteus mirabilis*	*Proteus mirabilis*	*Proteus mirabilis*	*Proteus mirabilis*	*Proteus mirabilis*
7	Mixed gram-positive flora	*Staphylococcus epidermidis*	*Staphylococcus epidermidis*	*Staphylococcus epidermidis*	*Staphylococcus epidermidis*
10	*Staphylococcus epidermidis*	*Yeast*	*Yeast*	*Candida parapsilosis*	*Candida parapsilosis*
11	*Escherichia coli*	*Yeast*	*Yeast*	*Candida albicans*	No growth
13	*Proteus mirabilis*	Mixed gram-positive flora Mixed gram-negative flora	*Proteus mirabilis Yeast*	*Candida tropicalis*	*Proteus mirabilis* *Candida tropicalis*
15	*Staphylococcus lugdunensis*	No growth	No growth	*Staphylococcus lugdunensis*	*Staphylococcus lugdunensis*
16	*Klebsiella oxytoca*	No culture performed	No growth	*Klebsiella oxytoca*	No growth
17	Mixed gram-positive flora	Mixed gram-positive flora	*Enterococcus faecalis*	*Enterococcus faecalis*	*Enterococcus faecalis*
18	*Acinetobacter baumannii* complex *Staphylococcus aureus*	No growth	*Yeast*	*Enterococcus faecalis*	No growth
20	Mixed gram-negative flora	Gram-negative rods	*Pseudomonas aeruginosa* Mixed gram-positive flora	*Enterococcus faecalis* *Citrobacter koseri*	*Enterococcus faecalis* *Citrobacter koseri*
21	*Staphylococcus aureus* *Enterococcus sp*.	Gram-positive cocci	No culture performed	*Staphylococcus aureus*	No growth
27	*Klebsiella pneumoniae* *Pseudomonas aeruginosa*	*Pseudomonas aeruginosa*	*Pseudomonas aeruginosa*	*Pseudomonas aeruginosa*	*Pseudomonas aeruginosa*
32	*Klebsiella pneumoniae*	No growth	No growth	*Klebsiella pneumoniae*	No growth
37	*Citrobacter freundii* complex	*Proteus mirabilis* *Enterococcus faecalis*	*Citrobacter freundii* complex *Enterococcus faecalis*	*Proteus mirabilis*	*Proteus mirabilis*
40	*Staphylococcus epidermidis*	*Staphylococcus epidermidis*	*Staphylococcus epidermidis*	*Staphylococcus epidermidis*	No growth
44	Mixed gram-positive flora Mixed gram-negative flora	*Proteus mirabilis* *Klebsiella pneumoniae* *Enterococcus faecalis*	*Enterococcus faecalis* *Morganella morganii*	*Proteus mirabilis* *Klebsiella pneumoniae*	*Proteus mirabilis* *Klebsiella pneumoniae*
46	*Klebsiella pneumoniae*	No growth	No growth	*Enterococcus faecalis* *Staphylococcus aureus*	*Enterococcus faecalis* *Staphylococcus aureus*
48	*Proteus mirabilis*	*Proteus mirabilis* *Pseudomonas aeruginosa*	*Proteus mirabilis* *Pseudomonas aeruginosa*	*Proteus mirabilis*	*Proteus mirabilis*
51	*Enterococcus faecalis*	*Enterobacter cloacae* complex	*Enterobacter cloacae* complex	*Enterococcus faecalis*	*Enterococcus faecalis*
53	No growth	*Escherichia coli*	*Escherichia coli*	*Escherichia coli*	*Escherichia coli*
54	No growth	No growth	No growth	*Pseudomonas fluorescens*	No growth
58	*Enterococcus faecium* *Stenotrophomonas maltophilia*	*Enterococcus faecium*	*Enterococcus faecium*	*Enterococcus faecium* *Stenotrophomonas maltophilia*	*Enterococcus faecium* *Stenotrophomonas maltophilia*
59	*Enterococcus* sp.	No growth	No growth	*Enterococcus faecalis*	*Enterococcus faecalis*
60	*Escherichia coli*	*Enterococcus faecalis*	*Enterococcus faecalis*	*Enterococcus faecalis*	*Enterococcus faecalis*

**TABLE 3:** Comparison of organism species exclusively seen with bead beating and mortar pestle

Sample no.	Bead beating			Mortar pestle		
	Mass (g)	Culture result	CFU/mL	Mass (g)	Culture result	CFU/mL
11	0.158	*Candida albicans*	100–1,000	0.197	*–*	–
13	0.135	*Candida tropicalis*	1,000–10,000	0.058	*Candida tropicalis* *Proteus mirabilis*	1,000–10,000 1,000–10,000
16	0.482	*Klebsiella oxytoca*	100–1,000	0.237	*–*	–
18	0.264	*Enterococcus faecalis*	1,000–10,000	0.151	***–***	–
21	0.200	*Staphylococcus aureus*	100–1,000	0.137	*–*	–
32	0.102	*Klebsiella pneumoniae*	100–1,000	0.201	*–*	–
40	0.271	*Staphylococcus epidermidis*	50,000–100,000	0.197	*–*	–
54	0.186	*Pseudomonas fluorescens*	>100,000	0.149	*–*	–

## DISCUSSION

In this study, we demonstrated that processing of PCNL-collected kidney stones via BB provided a statistically significantly higher rate of microbial detection compared to the traditional MP method. Specifically, BB was able to recover growth in 25 stones, while MP recovered growth in 18 stones. This suggests that BB potentially offers improved microbial recovery and superior sensitivity relative to MP, likely due to enhanced homogenization of stones prior to conducting stone culture. Further study is needed to explore whether BB can be adopted clinically as a preferred method for kidney stone cultures. Irrespective of the processing method, gram-negative and gram-positive bacteria were isolated from kidney stone specimens, along with *Candida* sp.

Compared to previous studies, our results demonstrate a heterogeneity of microbes, with *Enterococcus*, *Proteus*, *Klebsiella*, and *Staphylococcus* being the most common organisms isolated (Fig. 2). Furthermore, this was true among those stones in which BB showed exclusive growth, with *Klebsiella*, *Staphylococcus*, *Enterococcus*, and *Pseudomonas* among those isolated (Tables 2 and 3). Overall, the organisms identified in our study compare similarly to a previous meta-analysis, which found *Staphylococcus*, *Aerococcus*, *Enterobacterales*, and *Enterococcus* as the predominant groups (16).

The use of BB to homogenize tissue specimens is an established technique in microbiology that has been previously studied in diagnosing periprosthetic joint infections (PJI). This method has proven to be superior at facilitating bacterial release in PJI compared to other homogenization techniques (11, 13, 14) and has also been shown to be effective in the homogenization of mouse tissue samples (15). In the context of kidney stone cultures, however, BB is a relatively novel technique. To our knowledge, it has only been applied to assess kidney stone formation in artificial urine inoculated with bacteria (17).

A significant challenge in kidney stone microbiology is the lack of standardized protocols for stone culture processing (8). Variability in methodologies—including differences in stone homogenization techniques, culture media used, incubation times, and microbial identification methods—can lead to inconsistent microbial detection rates. This lack of standardization not only hampers the comparison of results across different studies but also affects clinical decision-making based on culture outcomes. With stone cultures during PCNL now recommended in EAU Guidelines (7), there is an urgent need for developing and adopting standardized, optimized methodologies to improve the reliability and reproducibility of microbial detection from kidney stones.

To our knowledge, this is the first study to compare BB to the traditional MP technique for kidney stone cultures. By providing a direct comparison, we offer evidence that BB is not only more efficient but also potentially more sensitive in detecting microbes from stone cultures. Additionally, the use of MALDI-TOF MS allowed for fast and highly specific identification of microorganisms, ensuring accurate results in a clinically relevant timeframe. This approach provided a robust means for microbial recovery and identification, making the findings both applicable and potentially useful for clinical microbiology.

Despite these strengths, our study has several limitations. The sample size of 60 stones, with bacterial growth in only 25 stones, may not fully capture the range of microbial diversity encountered in all cases of nephrolithiasis. Additionally, while we demonstrated an advantage of BB over MP in microbial recovery, further studies are needed to assess the clinical implications of these findings. Despite showing a propensity for BB-processed stones to have growth of organisms more often than MP-processed stones (Tables 2 and 3), there were a few notable differences in the weights of stones used to set up these cultures. Of the eight stones listed in Table 3 that had discordant results between BB and MP processing, six of the BB-processed stone fragments were heavier than those used for MP processing. This highlights the practical challenges of taking stones directly from the patient and studying them without additional manipulation—having stones of exactly the same weight would require an additional processing step of fragmentation, which even then is not guaranteed to result in exactly the same weighted stones. Furthermore, it is notable that in the two cases where the MP-processed fragment was heavier than the BB-processed fragment, the BB-associated culture was positive, whereas the MP-associated culture was not. Finally, it is unlikely that the organisms recovered in these cultures represent the full diversity of organisms within the stones (18), despite culturing these processed specimens similarly to high-quality/surgically collected urine specimens and enhancing incubation with 5% CO_2_.

Future work should evaluate whether BB-detected microbes correlate with clinical outcomes, such as postoperative severe infections—either systemic inflammatory response syndrome or quick Sequential Organ Failure Assessment. Moreover, the impact of antibiotic use prior to surgery may have influenced microbial recovery, as 19 out of 25 (76%) patients who had positive stone cultures had already received antibiotics preoperatively. Given that it is standard practice to treat a patient with bacteria detected on preoperative urine cultures, this factor needs to be explored in future studies to better understand the relationship between preoperative antibiotic use and microbial detection.

## CONCLUSIONS

BB is a more sensitive stone culture protocol in detecting bacteria within kidney stones when compared to the traditional technique of MP. As stone cultures are now recommended in Urology Guidelines, standardizing the methods by which these tests are performed will allow for improved data quality and a better understanding of how specific bacteria impact clinical outcomes.

## References

[1] Assimos D, Krambeck A, Miller NL, Monga M, Murad MH, Nelson CP, Pace KT, Pais VM, Pearle MS, Preminger GM, Razvi H, Shah O, Matlaga BR. 2016. Surgical management of stones: American Urological Association/Endourological Society Guideline, part II. J Urol 196:1161–1169. doi:10.1016/j.juro.2016.05.091 27238615

[2] Whitehurst L, Jones P, Somani BK. 2019. Mortality from Kidney Stone Disease (KSD) as reported in the literature over the last two decades: a systematic review. World J Urol 37:759–776. doi:10.1007/s00345-018-2424-2 30151599

[3] Zhou G, Zhou Y, Chen R, Wang D, Zhou S, Zhong J, Zhao Y, Wan C, Yang B, Xu J, Geng E, Li G, Huang Y, Liu H, Liu J. 2022. The influencing factors of infectious complications after percutaneous nephrolithotomy: a systematic review and meta-analysis. Urolithiasis 51:17. doi:10.1007/s00240-022-01376-5 36515726 PMC9750925

[4] Mariappan P, Smith G, Bariol SV, Moussa SA, Tolley DA. 2005. Stone and pelvic urine culture and sensitivity are better than bladder urine as predictors of urosepsis following percutaneous nephrolithotomy: a prospective clinical study. J Urol 173:1610–1614. doi:10.1097/01.ju.0000154350.78826.96 15821509

[5] Charton M, Vallancien G, Veillon B, Brisset JM. 1986. Urinary tract infection in percutaneous surgery for renal calculi. J Urol 135:15–17. doi:10.1016/s0022-5347(17)45500-5 3510316

[6] Korets R, Graversen JA, Kates M, Mues AC, Gupta M. 2011. Post-percutaneous nephrolithotomy systemic inflammatory response: a prospective analysis of preoperative urine, renal pelvic urine and stone cultures. J Urol 186:1899–1903. doi:10.1016/j.juro.2011.06.064 21944106

[7] Skolarikos A, Hung H, Neisius A, Petřík B, Somani T, Gambaro G, Tailly T. 2023. EAU guidelines on urolithiasis – limited. Update March 2023

[8] Tzou DT, Stern KL, Duty BD, Hsi RS, Canvasser NE, De S, Wong AC, Royal CR, Sloss ML, Ziemba JB, et al.. 2023. Heterogeneity in stone culture protocols and endourologist practice patterns: a multi-institutional survey. Urolithiasis 51:15. doi:10.1007/s00240-022-01373-8 36507964

[9] Yarbrough ML, Potter RF. 2022. Chapter 3.12.2 – Stone cultures. In Clinical microbiology procedures handbook, 4th ed. ASM Press, Washington DC.

[10] Redanz S, Podbielski A, Warnke P. 2015. Improved microbiological diagnostic due to utilization of a high-throughput homogenizer for routine tissue processing. Diagn Microbiol Infect Dis 82:189–193. doi:10.1016/j.diagmicrobio.2015.03.018 25886816

[11] Roux A-L, Sivadon-Tardy V, Bauer T, Lortat-Jacob A, Herrmann J-L, Gaillard J-L, Rottman M. 2011. Diagnosis of prosthetic joint infection by beadmill processing of a periprosthetic specimen. Clin Microbiol Infect 17:447–450. doi:10.1111/j.1469-0691.2010.03359.x 20825439

[12] Drago L, Clerici P, Morelli I, Ashok J, Benzakour T, Bozhkova S, Alizadeh C, Del Sel H, Sharma HK, Peel T, Mattina R, Romanò CL. 2019. The World Association against Infection in Orthopaedics and Trauma (WAIOT) procedures for microbiological sampling and processing for Periprosthetic Joint Infections (PJIs) and other implant-related infections. J Clin Med 8:933. doi:10.3390/jcm8070933 31261744 PMC6678965

[13] Jazmati N, Liebold C, Offerhaus C, Volkenand A, Grote S, Pöpsel J, Körber-Irrgang B, Hoppe T, Wisplinghoff H. 2024. Rapid high-throughput processing of tissue samples for microbiological diagnosis of periprosthetic joint infections using bead-beating homogenization. J Clin Microbiol 62:e0148623. doi:10.1128/jcm.01486-23 38415637 PMC11005376

[14] Cai Y, Fang X, Zhang L, Yang X, Nie L, Huang Z, Li W, Zhang C, Yang B, Guan Z, Zhang W. 2021. Microbial yield from infectious tissues pretreated by various methods: an invitro study. BMC Musculoskelet Disord 22:209. doi:10.1186/s12891-021-04071-5 33612121 PMC7898421

[15] Liang X, Ubhayakar S, Liederer BM, Dean B, Ran-Ran Qin A, Shahidi-Latham S, Deng Y. 2011. Evaluation of homogenization techniques for the preparation of mouse tissue samples to support drug discovery. Bioanalysis 3:1923–1933. doi:10.4155/bio.11.181 21899502

[16] Kachroo N, Lange D, Penniston KL, Stern J, Tasian G, Bajic P, Wolfe AJ, Suryavanshi M, Ticinesi A, Meschi T, Monga M, Miller AW. 2021. Meta-analysis of clinical microbiome studies in urolithiasis reveal age, stone composition, and study location as the predominant factors in urolithiasis-associated microbiome composition. mBio 12:e0200721. doi:10.1128/mBio.02007-21 34372696 PMC8406293

[17] Wallace B, Chmiel JA, Al KF, Bjazevic J, Burton JP, Goldberg HA, Razvi H. 2023. The role of urinary modulators in the development of infectious kidney stones. J Endourol 37:358–366. doi:10.1089/end.2022.0458 36562270

[18] Moreland RB, Brubaker L, Tinawi L, Wolfe AJ. 2025. Rapid and accurate testing for urinary tract infection: new clothes for the emperor. Clin Microbiol Rev 38:e0012924. doi:10.1128/cmr.00129-24 40366169 PMC12160498

